# Exploration of JAK/STAT pathway activation in ulcerative colitis reveals sex-dependent activation of JAK2/STAT3 in the inflammatory response

**DOI:** 10.3389/fimmu.2025.1609740

**Published:** 2025-07-21

**Authors:** Cristina Calviño-Suárez, Mariña Durán-Rubí, José Brea, David Moreira, Inés Ardao, Iria Brocos-Mosquera, Rocío Ferreiro-Iglesias, Sol Porto-Silva, Laura Nieto-Garcia, María José Varela, María Isabel Loza, Antón L. Martínez, Manuel Barreiro-de Acosta

**Affiliations:** ^1^ Department of Gastroenterology and Hepatology, University Hospital of Santiago De Compostela, Santiago de Compostela, Spain; ^2^ Instituto de Investigacións Sanitarias de Santiago de Compostela (IDIS), Santiago de Compostela, Spain; ^3^ Innopharma Drug Screening and Pharmacogenomics Platform, BioFarma Research Group, Center for Research in Molecular Medicine and Chronic Diseases (CiMUS), Department of Pharmacology, Pharmacy and Pharmaceutical Technology, Faculty of Pharmacy, University of Santiago de Compostela, Santiago de Compostela, Spain; ^4^ Kaertor Foundation, Santiago de Compostela, Spain

**Keywords:** ulcerative colitis, JAK/STAT pathway, phosphorylation, sex-specific differences, inflammatory signaling, personalized therapy

## Abstract

**Introduction:**

Ulcerative colitis (UC) is characterized by aberrant immune responses involving multiple inflammatory pathways, including JAK/STAT signaling. However, the specific roles and interactions of individual components within this pathway remain unclear.

**Methods:**

We conducted a prospective, observational, single-center study enrolling 61 adult UC patients undergoing routine colonoscopy with endoscopic activity (Mayo Endoscopic Score > 0). Paired biopsies from inflamed and non-inflamed colonic mucosa were collected. Phosphorylation levels of JAK1, JAK2, JAK3, TYK2, STAT1, STAT3, and STAT4 were quantified by Western blot.

**Results:**

Inflamed tissue showed significantly increased phosphorylation of JAK2, JAK3, TYK2, STAT1, STAT3, and STAT4 compared to non-inflamed mucosa (p < 0.05), while JAK1 levels did not differ significantly. Correlation analysis revealed coordinated activation among JAK2, JAK3, TYK2, and STAT3, suggesting interdependent roles. Notably, male patients exhibited significantly higher activation of JAK2 and STAT3 than female patients (p < 0.05).

**Discussion:**

These findings highlight a heterogeneous but important involvement of the JAK/STAT pathway in UC pathophysiology. The observed sex-specific differences and coordinated activation patterns suggest the value of personalized therapeutic approaches targeting specific components of this pathway.

## 1 Introduction

Inflammatory bowel disease (IBD) encompasses two primary conditions: ulcerative colitis (UC) and Crohn’s disease (1). UC is a chronic inflammatory disorder characterized by inflammation of the colonic mucosa, typically beginning in the rectum and extending proximally. Its diagnosis hinges on endoscopic and histopathological evidence of colonic inflammation (2). UC follows an unpredictable course, characterized by periods of relapse and remission, which significantly impact patient quality of life and place a substantial burden on healthcare systems (3).

The etiology of UC remains elusive, but current understanding suggests it arises from complex interactions among the gut microbiota, genetics, and various immunological and environmental factors (4). UC affects approximately 1% of the population in some industrialized countries (5) and its prevalence has notably increased in newly industrialized nations in recent years (6), highlighting lifestyle factors as potential risk factors and linking environmental triggers to immune dysregulation. Despite recent advances in UC management and the availability of multiple therapeutic options targeting various inflammatory signaling pathways, the disease remains incurable. A significant proportion of patients experience limited or no response to treatment, resulting in overtreatment, unnecessary exposure to side effects, and delays in receiving optimal therapeutic interventions. Additionally, treatment response is highly variable among patients with similar clinical presentations (7–10), emphasizing the critical need for predictive biomarkers of therapeutic efficacy. Developing tools that link the activation of specific inflammatory pathways with individual patient responses could facilitate personalized treatment strategies, improving therapeutic outcomes and reducing the rate of non-responders.

Central to UC’s pathogenesis is an aberrant immune response, with several inflammatory pathways, including the JAK/STAT signaling pathway, playing crucial roles (11, 12). The Janus kinase (JAK) family—comprising JAK1, JAK2, JAK3, and tyrosine kinase 2 (TYK2)—mediates cytokine signaling by phosphorylating and activating signal transducer and activator of transcription (STAT) proteins. Once phosphorylated, STAT proteins dimerize and translocate to the nucleus, where they regulate the transcription of genes involved in immune responses and inflammation (12). Overactivation of this pathway can result in a proinflammatory state, driving the chronic inflammation and tissue damage characteristic of UC (13). This dysregulation is reflected in abnormal phosphorylation levels, further perpetuating immune activation (14). This disrupts cytokine signaling, driving the excessive immune activation and chronic inflammation characteristic of UC (13, 15). Various cytokines, such as IFNγ, IL-6, IL-12, IL-15 and IL-23, signal through this pathway to modulate immune responses and contribute to intestinal tissue damage (16), thus playing a central role in UC pathogenesis (12, 17). For example, IL-6 disrupts the intestinal barrier and promotes immune cell infiltration via STAT3 activation (18). These observations underscore the central role of the JAK/STAT pathway in orchestrating inflammatory responses in UC.

Further complicating UC management is the significant heterogeneity observed in disease presentation, complications, and treatment response among patients (19). Increasing evidence indicates that sex-specific factors, including hormonal and genetic differences, modulate inflammatory pathways, contributing to variations between males and females in clinical outcomes such as mortality, surgery rates, and comorbidities (20, 21). Notably, differential immune activation patterns between sexes underscore the importance of incorporating sex as a biological variable in UC research and personalized therapeutic approaches (22, 23). A deeper understanding of these sex-specific influences may improve predictive accuracy for treatment response and facilitate the development of tailored therapeutic strategies.

Thus, our working hypothesis was that profiling the activation of the JAK/STAT inflammatory pathway in UC patients could provide valuable insights into the pathophysiology of UC and help elucidate the variability observed among patients. To address this, our objectives were: i) to analyze the activation of JAK/STAT pathway components in colon samples of a cohort of UC patients; ii) to establish correlations among activation levels of these proteins, revealing interconnections relevant to UC pathophysiology; and iii) to investigate sex-specific differences in pathway activation. These explorations aim to enhance our understanding of UC pathophysiology and lay the groundwork for developing personalized therapeutic strategies in UC.

## 2 Materials and methods

### 2.1 Study design and population

A prospective, observational single-center study was designed. Adult UC patients exhibiting any level of endoscopic activity [Mayo Endoscopic Score (MES) > 0] during routine or diagnostic colonoscopy were included. Exclusion criteria encompassed patients who had undergone colonic or other abdominal surgeries within the past six months, those diagnosed with infectious colitis or HIV, patients with other immune-mediated diseases besides UC, individuals with unresolved malignancies or those who were pregnant. The study was approved by the Galician Ethics Committee on Drug Research (CEIm-G) and was conducted in accordance with the local legislation and institutional requirements. All the participants provided their written informed consent prior to the colonoscopy and biopsy collection.

### 2.2 Patient data and sample collection

Patient data were extracted from electronic medical records and recorded in a case report form. Collected information included sex, date of birth, UC diagnosis date, disease extension (Montreal classification) at diagnosis, current endoscopic activity (MES), fecal calprotectin (FC) levels before colonoscopy and history of UC-related medications.

During routine clinical colonoscopies, three biopsies were taken from colonic mucosa with endoscopic activity (MES > 0) and three from areas without endoscopic activity (MES = 0) for each patient. All samples were pseudo-anonymized and stored at -80°C until processed.

### 2.3 Sample processing

Colon samples underwent mechanical digestion and sonication in a lysis buffer containing 50 mM Tris (T1503; Merck, Madrid, Spain), 150 mM NaCl (31434; Merck), 5 mM EDTA (ED2SS; Merck) and supplemented with Complete EDTA-free Protease Inhibitor (11873580001; Merck) and PhosSTOP (4906845001; Merck). Post-digestion, the samples were centrifuged for 10 minutes at 10000 x g and the protein concentration was determined using the RD DC protein assay kit (5000121; Bio-Rad, Alcobendas, Madrid, Spain). The extracts were then stored at -80°C until further analysis.

### 2.4 Determination of JAK/STAT pathway activation

Activation of the JAK/STAT pathway was analyzed by Western blot. Aliquots containing 20 µg of total protein extracted from colonic biopsies were mixed with Bolt LDS sample buffer (B007; Thermo-Fisher Scientific, Alcobendas, Madrid, Spain) and heated to 95°C for 5 minutes. Proteins were then separated on Bolt 4–12% Bis-Tris Plus gels (NW4127; Thermo-Fisher Scientific) at 120 V for 85 minutes and subsequently transferred onto 0.2 μm PVDF membranes (1620177; BioRad) at 15 V for 90 minutes.

After transfer, membranes were treated with SuperSignal Western Blot Enhancer (10157958; Thermo-Fisher Scientific), blocked in 5% bovine serum albumin (BSA; 10775835001; Merck), and incubated overnight (16 h) at 4°C with the corresponding primary antibodies listed in **Table 1**. After washing, membranes were incubated at room temperature with a secondary anti-rabbit HRP-linked antibody (7074; Thermo-Fisher Scientific) at the dilutions specified in **Table 1**. All phospho-specific antibodies used in this study were commercially available and validated by the manufacturer using defined positive control lysates. These lysates, derived from recommended cell lines, were also used in-house before initiating patient sample analysis to confirm signal specificity and expected molecular weight under our experimental conditions.

**Table 1:** Conditions for the semiquantification of the phosphorylation of the proteins in JAK/STAT pathway.

Protein	Primary antibody		Secondary Antibody	Manufacturer’s validation cell line
	Reference and provider	Dilution	Dilution	
JAK1P	44-422G; Thermo-Fisher Scientific	1:400	1:7500	CAL27 treated with IL-6
JAK2P	710928; Thermo-Fisher Scientific	1:500	1:7500	HT-29 treated with MG-132 and IFN-γ
JAK3P	PA5-40264; Thermo-Fisher Scientific	1:400	1:7500	Jurkat treated with IL-15
TYK2P	PA5-37762; Thermo-Fisher Scientific	1:1300	1:5000	HT29 cells (positive control)
STAT1P	ab109457; Abcam	1:1000	1:3000	A431 treated with EGF
STAT3P	ab76315; Abcam	1:1500	1:5000	HeLa treated with IFNα
STAT4P	4134; Cell Signaling	1:750	1:7500	NK-92 treated with IL-2

Protein detection was performed using the Pierce ECL Western Blotting Substrate (RPN2232; Thermo-Fisher Scientific) and visualized on an iBright CL1500 imaging system (Thermo-Fisher Scientific). β-actin, detected using an anti-actin antibody coupled to HRP (1:100,000 dilution; 12262S; Cell Signaling, Leiden, Netherlands), served as loading control.

To allow sequential probing of multiple phosphorylated proteins on the same membrane, blots were subsequently stripped using Restore Western Blot Stripping Buffer (21059; Thermo-Fisher Scientific) for 10 minutes at room temperature. After stripping, membranes were re-blocked and re-incubated overnight at 4°C with a different primary antibody, followed by incubation with the secondary antibody for 1 hour at room temperature. Protein detection was repeated using the ECL substrate and iBright imaging system as previously described.

Quantitative band analysis was performed using ImageJ software version 1.53t (NIH, Bethesda, MD, USA) to calculate the integrated volume (average optical density of pixels within the object area per mm²) (24). The integrated intensity of each phosphorylated protein in non-inflamed areas was assigned a baseline value of 1, enabling comparative densitometric analysis of JAK/STAT pathway activation in inflamed mucosa. Results are expressed as mean ± standard deviation (S.D.) of the ratio of integrated band intensities (inflamed vs. non-inflamed areas) for each protein.

### 2.5 Data analysis

Phosphorylation levels of JAK/STAT pathway components were compared between inflamed and non-inflamed areas using one-way ANOVA with Sidak’s multiple comparisons test in GraphPad Prism 10 (GraphPad Software, La Jolla, CA, USA). Pearson correlation coefficients were calculated using R statistical software (version 4.3.3) (25), to assess relationships between JAK/STAT pathway components, and Mann-Whitney U tests were performed in GraphPad Prism 10 to evaluate sex-related differences. Normality of data distributions was assessed using the Shapiro-Wilk test, and appropriate parametric or non-parametric tests were applied. Results were visualized using heatmaps, bar plots, and box-and-whisker plots created with GraphPad Prism. Statistical significance was set at *p* < 0.05.

The *post hoc* statistical power of the Mann–Whitney U test was estimated by Monte Carlo simulation using the *wmwpow* package (version 0.1.3) in R software. Based on the observed data distributions in both groups, 10,000 simulated datasets were generated under the alternative hypothesis. For each simulated dataset, the Mann–Whitney U test was performed, and the proportion of simulations yielding a statistically significant result (p < 0.05) was computed to estimate the achieved power.

## 3 Results

### 3.1 Sixty-one patients with UC were included in the final analysis

**Figure 1:**
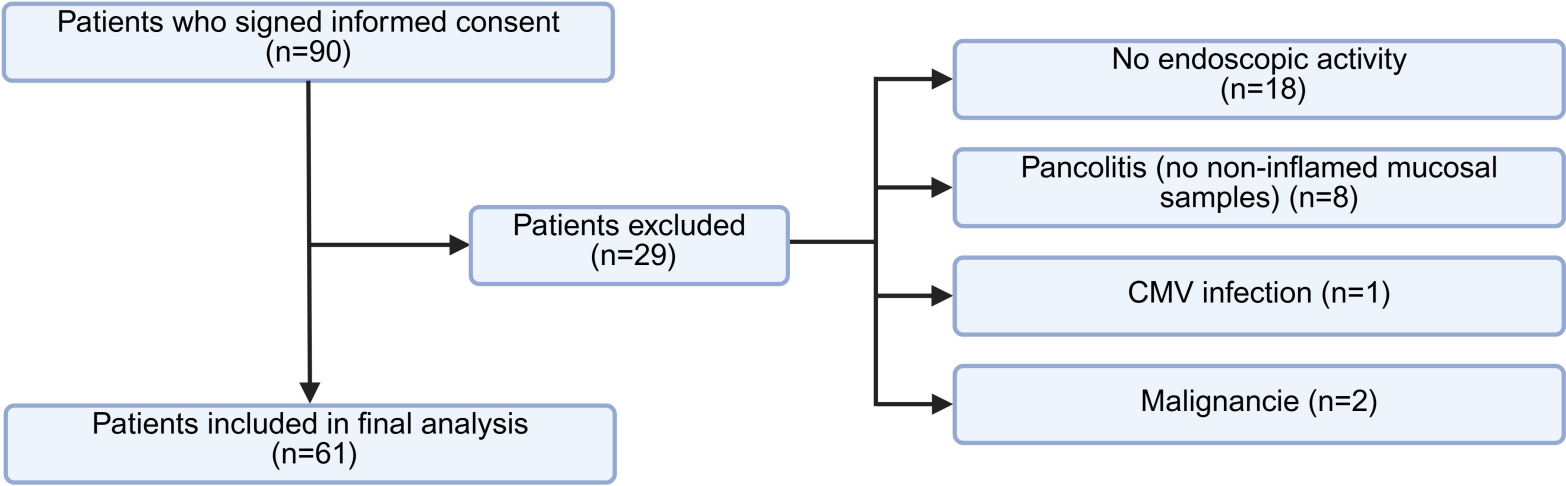
Flowchart of patient selection. Diagram illustrating the inclusion process of UC patients and reasons for exclusion. Figure created with BioRender.com.

The patient selection process is detailed in **Figure 1**. Of the ninety patients who signed informed consent, twenty-nine were excluded from the final analysis: eighteen due to absence of endoscopic activity, eight due to pancolitis without non-inflamed areas available for paired sampling, and three based on exclusion criteria—two due to previously undiagnosed malignancies identified upon clinical record review, and one due to active cytomegalovirus infection. Ultimately, sixty-one patients were included, each providing paired biopsies from inflamed and non-inflamed colonic mucosa.

The demographic and clinical characteristics of the included patients are summarized in **Table 2**. The cohort consisted of thirty-one female (50.8%) and thirty male patients (49.2%), with a median age of 52.9 years. Notably, six patients (9.8%) were treatment-naïve, while fifty-five (90.2%) were receiving treatment at the time of sample collection. Fifty-one patients (83.6%) had not previously received advanced therapies, whereas ten (16.4%) were under biologic treatment. Importantly, no patients were being treated with tofacitinib at the time of sampling, ensuring that the observed JAK/STAT pathway activation was not influenced by direct pharmacological JAK inhibition.

**Table 2:** Baseline characteristics of patients included in the analysis.

Variable	Category or value	n (%) or summary measure
**Sex**		n (%)
	Female	31 (50.8)
	Male	30 (49.9)
**Age (Years)**	Median (IQR)	52.94 (39.0-64.7)
**Disease duration (Years)**	Mean (S.D.)	11.2 (9.2)
**Age at diagnosis (Years)**	Mean (S.D.)	41.0 (14.3)
**Disease location**		n (%)
	Proctitis	17 (27.9)
	Left-side colitis	32 (52.5)
	Extensive colitis	12 (19.7)
**Mayo Endoscopic Score**		n (%)
	1	1 (1.6)
	2	46 (75.4)
	3	14 (23.0)
**Faecal Calprotectin**		n (%)
	<150 mg/kg	17 (27.9)
	≥150 mg/kg	40 (65.6)
	Unknown	4 (6.6)
**Treatment at the time of colonoscopy** ^a^		n (%)
	No treatment	6 (9.8)
	Oral 5-ASA	49 (80.3)
	Topical 5-ASA	60 (65.7)
	Steroids	11 (18.0)
	Azathioprine	6 (9.8)
	Methotrexate	7 (11.5)
	Anti-TNF	3 (4.9)
	Vedolizumab	3 (4.9)
	Ustekinumab	4 (6.6)
	Tofacitinib	0 (0)

a 46 patients (75.4%) were under more than one treatment at the time of colonoscopy.

### 3.2 An increase in the activation of proteins from the JAK/STAT pathway is observed in those areas with endoscopic activity of the colon of patients with UC

**Figure 2:**
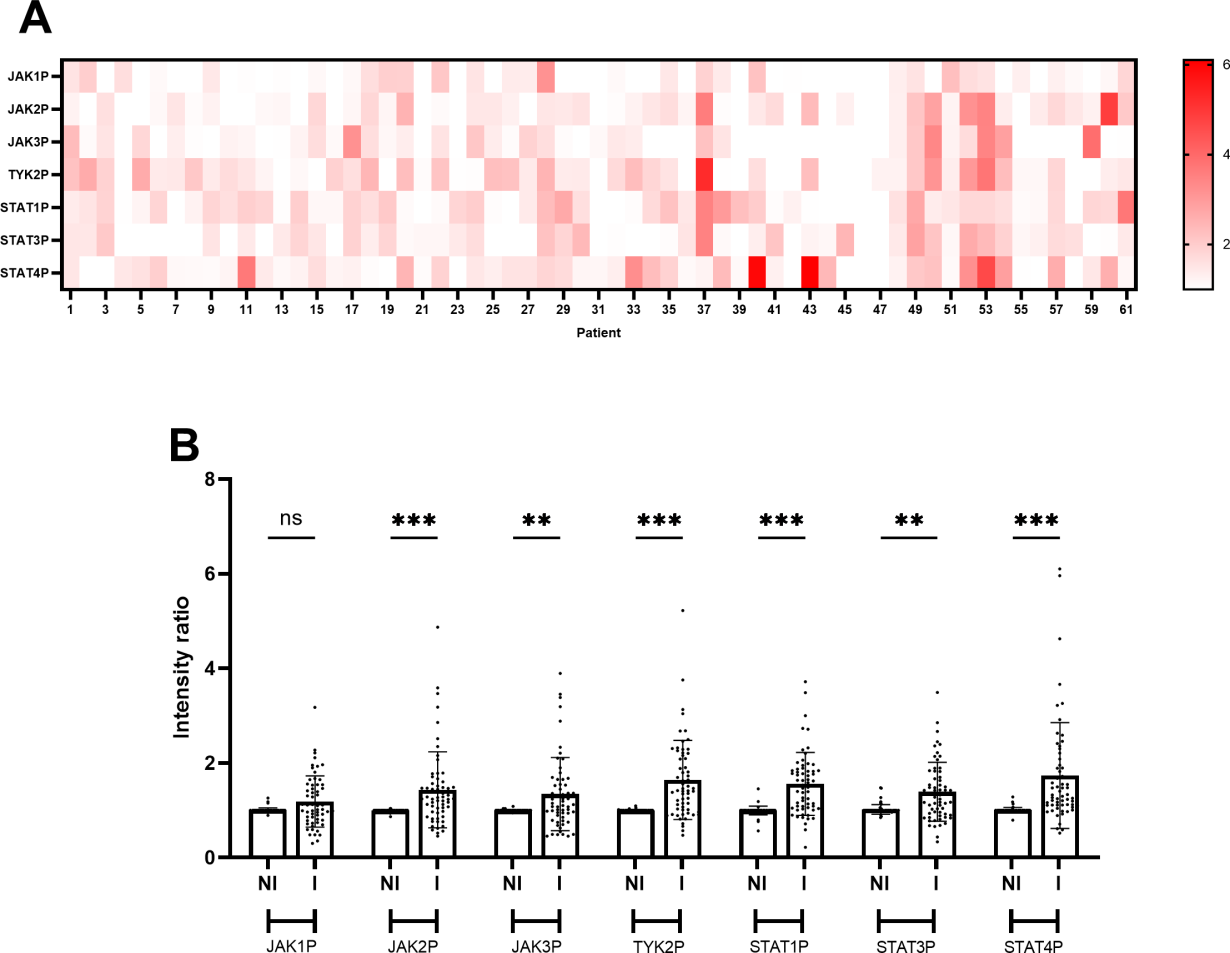
JAK/STAT pathway activation is higher in inflamed areas of the colon compared to non-inflamed areas in UC patients. **(A)** Heatmap illustrating phosphorylation levels of JAK/STAT pathway proteins in the sixty-one UC patients analyzed in the study. Data represent the mean values of the ratio between band intensities of phosphorylated JAK/STAT pathway components in inflamed areas of the colon compared with non-inflamed areas, derived from at least two independent experiments (N=2) each performed in duplicate points (n=2) for each of the sixty-one patients. **(B)** Bar plot comparing phosphorylation levels of JAK/STAT pathway components between non-inflamed (NI) and inflamed (I) colon areas in UC patients. Data represent the mean ± standard deviation of the ratio of band intensities for phosphorylated JAK/STAT pathway components in the sixty-one patients included in the study cohort, based on at least two independent experiments (N=2) each performed in duplicate points (n=2) for each of the sixty-one patients. Individual points represent values for each patient. Statistical analysis was performed using ANOVA with Sidak’s test; *n.s.*, non-significant; **p < 0.01; ***p < 0.001.

Variable activation patterns of the JAK/STAT pathway were observed in areas with endoscopic activity among the sixty-one UC patients included in the analysis (**Figure 2A**). A global comparison of the phosphorylation levels of all the analyzed proteins revealed a significant increase of JAK2, JAK3, TYK2, STAT1, STAT3, and STAT4 phosphorylation in areas with endoscopic activity compared to areas without endoscopic activity (p < 0.05; **Figure 2B**). In contrast, no significant difference was observed in the global phosphorylation levels of JAK1 between areas with and without endoscopic activity (*p* = 0.425; **Figure 2B**). Representative Western blot images for the seven phosphorylated JAK/STAT pathway components are provided in **Supplementary Figure 1**.

### 3.3 Correlation analysis revealed significant relationships between the phosphorylation levels of JAK/STAT pathway components in UC patients

**Figure 3:**
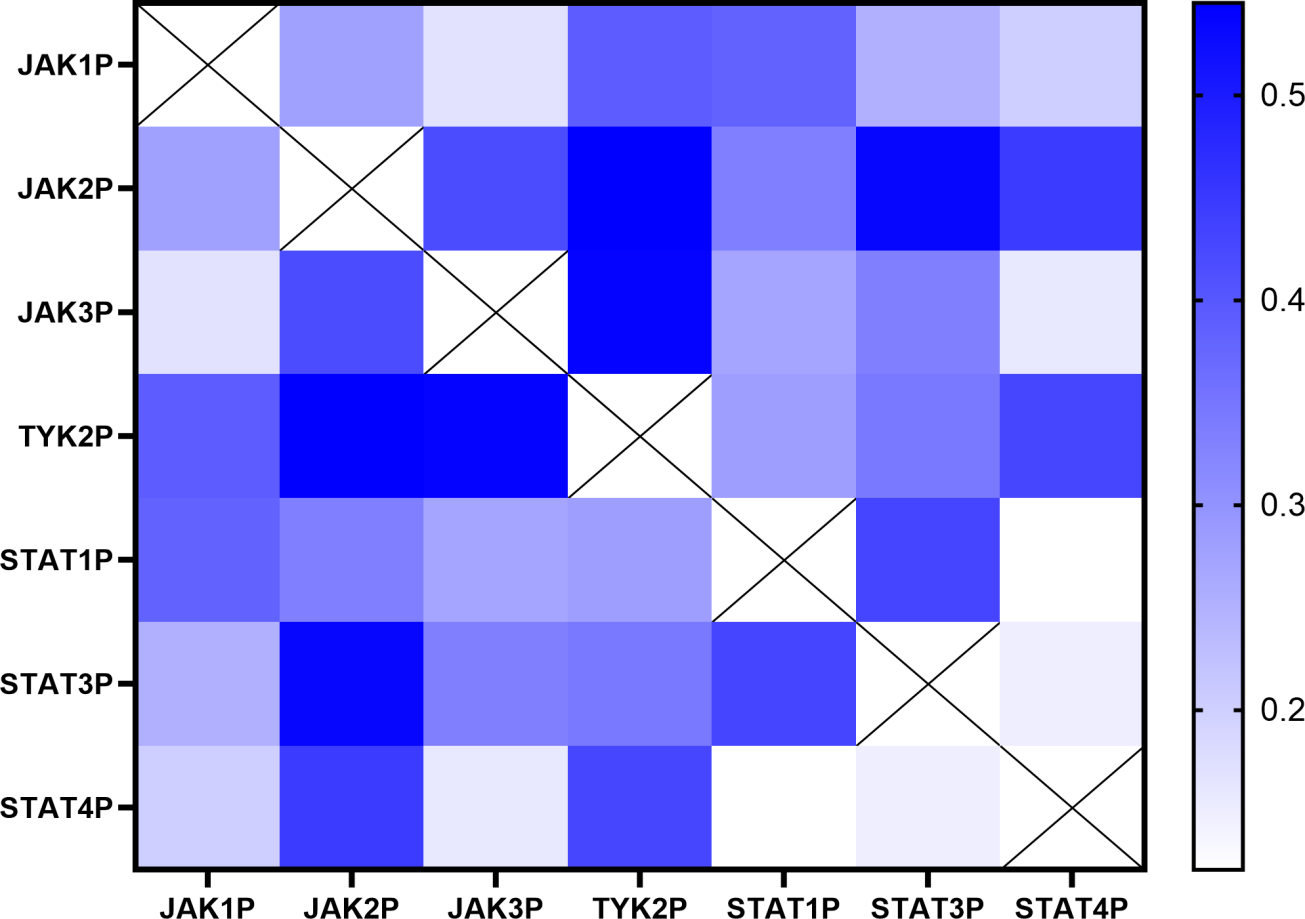
Correlation matrix of phosphorylation levels of JAK/STAT pathway components in UC patients. Heatmap illustrating the Pearson correlation coefficients between phosphorylation levels of JAK1, JAK2, JAK3, TYK2, STAT1, STAT3, and STAT4 in the sixty-one UC patients included in the study. The intensity and color of each cell represent the strength of the positive correlation (blue: stronger positive correlation; white: no correlation). The analysis was performed using data derived from at least two independent experiments (N=2) each performed in duplicate points (n=2) for each of the sixty-one patients.

To explore potential interactions within the JAK/STAT pathway, a correlation analysis was conducted using phosphorylation levels of JAK1, JAK2, JAK3, TYK2, STAT1, STAT3, and STAT4 across the cohort of sixty-one UC patients. The Pearson correlation coefficients are visualized in a heatmap, where the intensity and color of each cell represent the strength of positive correlations between pathway components (**Figure 3**). This analysis identified multiple significant correlations, highlighting coordinated patterns of activation within the JAK/STAT signaling pathway.

Detailed statistical results, including Pearson correlation coefficients, 95% confidence intervals, and significance levels for each pair of JAK/STAT pathway components, are summarized in **Table 3**. Strong positive correlations were observed between phosphorylation levels of JAK2 and TYK2 (*r* = 0.545, *p* < 0.001), JAK2 and STAT3 (*r* = 0.534, *p* < 0.001), and JAK3 and TYK2 (*r* = 0.539, *p* < 0.001). Weaker yet statistically significant correlations were identified between TYK2 and STAT4 (*r* = 0.428, *p* = 0.010), as well as between STAT1 and STAT3 (*r* = 0.429, *p* = 0.010). Collectively, these results suggest a coordinated pattern of activation among specific JAK/STAT pathway components in UC.

**Table 3:** Pearson correlation coefficients, confidence intervals, and significance levels for phosphorylation levels of JAK/STAT pathway components in UC patients.

Comparison	Correlation coefficient (*r*)	95% confidence interval (Lower-Upper)	Degrees of freedom	Significance level (*p*)^a^
**JAK1P-JAK2P**	0.279	0.029 - 0.496	59	0.251 (*n.s.*)
**JAK1P-JAK3P**	0.169	-0.086 - 0.404	59	0.767 (*n.s.*)
**JAK1P-TYK2P**	0.391	0.154 - 0.585	59	0.026 (*)
**JAK1P-STAT1P**	0.381	0.143 - 0.578	59	0.032 (*)
**JAK1P-STAT3P**	0.253	0.002 - 0.475	59	0.292 (*n.s.*)
**JAK1P-STAT4P**	0.201	-0.054 - 0.431	59	0.604 (*n.s.*)
**JAK2P-JAK3P**	0.418	0.186 - 0.606	59	0.012 (*)
**JAK2P-TYK2P**	0.545	0.339 - 0.700	59	< 0.001 (***)
**JAK2P-STAT1P**	0.333	0.089 - 0.540	59	0.096 (*n.s.*)
**JAK2P-STAT3P**	0.534	0.326 - 0.692	59	< 0.001 (***)
**JAK2P-STAT4P**	0.446	0.219 - 0.627	59	0.006 (**)
**JAK3P-TYK2P**	0.539	0.333 - 0.697	59	< 0.001 (***)
**JAK3P-STAT1P**	0.271	0.021 - 0.490	59	0.251 (*n.s.*)
**JAK3P-STAT3P**	0.332	0.088 - 0.539	59	0.096 (*n.s.*)
**JAK3P-STAT4P**	0.16	-0.096 - 0.396	59	0.767 (*n.s.*)
**TYK2P-STAT1P**	0.282	0.032 - 0.498	59	0.251 (*n.s.*)
**TYK2P-STAT3P**	0.344	0.101 - 0.549	59	0.079 (*n.s.*)
**TYK2P-STAT4P**	0.428	0.198 - 0.614	59	0.010 (*)
**STAT1P-STAT3P**	0.429	0.199 - 0.614	59	0.010 (*)
**STAT1P-STAT4P**	0.122	-0.134 - 0.363	59	0.767 (*n.s.*)
**STAT3P-STAT4P**	0.150	-0.106 - 0.387	59	0.767 (*n.s.*)

a Pearson correlation. Significance levels are denoted as follows: *p <* 0.05 (*), *p <* 0.01 (**), *p <* 0.001 (***); *n.s.*, non-significant.

Conversely, certain JAK/STAT pathway components, such as TYK2 and STAT1 (*r* = 0.282, *p* = 0.251) and STAT3 and STAT4 (*r* = 0.150, *p* = 0.767), exhibited no significant correlations in their activation, indicating that not all components of the pathway are equally interconnected in UC pathophysiology. This nuanced correlation profile underscores the complexity of the JAK/STAT pathway.

### 3.4 Sex-related differences in the phosphorylation levels of JAK/STAT pathway components were observed in UC patients

**Figure 4:**
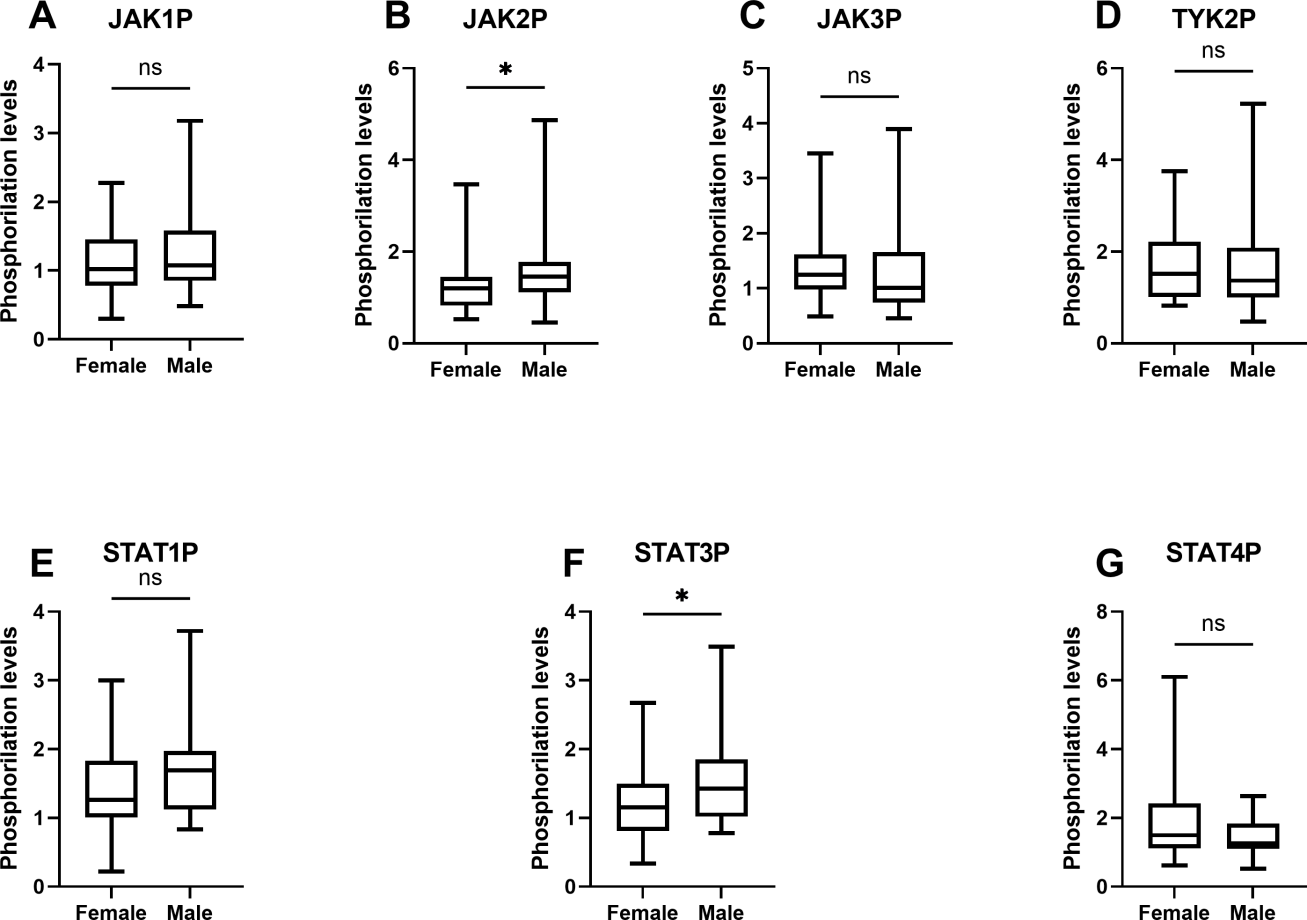
Box-and-whisker plots of phosphorylation levels of JAK/STAT pathway components in UC patients by sex. The plots illustrate the distribution of phosphorylation levels of **(A)** JAK1, **(B)** JAK2, **(C)** JAK3, **(D)** TYK2, **(E)** STAT1, **(F)** STAT3, and **(G)** STAT4 in male and female UC patients. The analysis was performed using data derived from at least two independent experiments (N=2) each performed in duplicate points (n=2) for each of the sixty-one patients. Data represent the median, interquartile range (IQR), and minimum and maximum values, analyzed using the Mann-Whitney U test. Significant differences are indicated (*p < 0.05).

The activation intensity of JAK/STAT pathway components in UC patients, stratified by sex, is summarized in **Table 4** and illustrated using box-and-whisker plots in **Figure 4**. Significant differences in phosphorylation levels were observed for JAK2 (*p* = 0.049) and STAT3 (*p* = 0.033), suggesting a potential sex-related influence on the activation of these specific proteins. In contrast, no significant differences were found for JAK1 (*p* = 0.743), JAK3 (*p* = 0.170), TYK2 (*p* = 0.475), STAT1 (*p* = 0.076) or STAT4 (*p* = 0.240), suggesting that sex may not uniformly influence phosphorylation patterns within the JAK/STAT pathway. *Post hoc* power analyses indicated that the observed sex-related differences in STAT3 and JAK2 phosphorylation achieved statistical powers of 0.573 and 0.505, respectively. For the remaining proteins, statistical power values ranged from 0.065 to 0.425 (**Table 4**), suggesting limited sensitivity to detect subtle sex-related differences in the current sample size.

**Table 4:** Mean and interquartile range (IQR) intensity of phosphorylated JAK/STAT pathway components in female and male UC patients, with statistical comparisons and power values.

Protein	Females		Males		Significance level (*p*)^a^	Power (Mann-Whitney)
	Median intensity	Interquartile rank (IQR)	Median intensity	Interquartile rank (IQR)		
**JAK1P**	1.022	0.783 - 1.457	1.073	0.850 - 1.580	0.745 (*n.s.*)	0.065
**JAK2P**	1.200	0.822 - 1.447	1.454	1.113 - 1.780	0.049 (*)	0.505
**JAK3P**	1.247	0.977 - 1.613	1.010	0.739 - 1.656	0.170 (*n.s.*)	0.287
**TYK2P**	1.513	1.010 - 2.216	1.366	1.003 - 2.088	0.475 (*n.s.*)	0.425
**STAT1P**	1.265	1.006 - 1.827	1.692	1.124 - 1.973	0.077 (*n.s.*)	0.573
**STAT3P**	1.153	0.811 - 1.499	1.429	1.020 - 1.853	0.033 (*)	0.22
**STAT4P**	1.498	1.114 - 2.414	1.264	1.094 - 1.839	0.240 (*n.s.*)	0.114

a Mann-Whitney U Test. Significance levels are denoted as follows: *p < 0.05; *n.s.*, non-significant.

## 4 Discussion

The primary achievement of this study was the comprehensive profiling of JAK/STAT pathway activation in samples of inflamed and non-inflamed colonic areas from a cohort of sixty-one patients with UC. This approach allowed us to: i) demonstrate a significant increase in the phosphorylation of six of the seven JAK/STAT pathway components analyzed (JAK2, JAK3, TYK2, STAT1, STAT3, and STAT4) in the inflamed colonic areas of UC patients; ii) identify interconnections in the activation of JAK2, JAK3, TYK2 and STAT3 proteins within the JAK/STAT pathway; and iii) uncover a sex-dependent activation of JAK2 and STAT3. These findings align with our hypothesis, providing valuable insights into the pathophysiology of UC, addressing the observed variability among patients, and establishing a foundation for developing novel diagnostic tools and personalized therapeutic strategies.

The demographic and clinical features of our cohort are representative of real-world UC patient populations, aligning well with data from previous studies (26), and providing a robust framework for investigating the activation of the JAK/STAT pathway in UC. Importantly, the majority of patients included had not received advanced therapies such as biologics, and none were treated with tofacitinib or other small-molecule JAK inhibitors at the time of sampling, ensuring that observed patterns of JAK/STAT activation were not influenced by direct pharmacological inhibition. Moreover, the inclusion of six treatment-naïve patients (9.8%) further enhances the representativeness and generalizability of our findings.

Although considerable variability in the phosphorylation levels of JAK/STAT pathway components was observed among the patients included in the analysis, significant increases were detected in the phosphorylation of JAK2, JAK3, TYK2, STAT1, STAT3, and STAT4 in inflamed colonic samples of UC patients compared to non-inflamed samples. In contrast, no significant changes were observed in JAK1 phosphorylation.

The observed increase in the phosphorylation of JAK2, JAK3, and TYK2 in our cohort aligns closely with findings in the existing literature, which highlight their critical roles in the pathophysiology of UC. JAK2 is a key mediator in the signaling pathways of several cytokines involved in UC-related inflammation, including IL-6, IL-12 and IL-23 (27, 28). Similarly, JAK3 is involved in the signaling pathways of cytokines relevant to UC, such as IL-15 (16, 17), further supporting its role in intestinal inflammation as documented in prior studies (29). TYK2, which plays a pivotal role in the signaling of IL-12 and IL-23 (27, 30), is also recognized as a key driver of chronic inflammation in UC (31). Thus, our data corroborate these findings, further reinforcing the importance of JAK2, JAK3, and TYK2 as central mediators in UC-associated inflammation as described previously in the literature.

The increased phosphorylation of STAT1, STAT3, and STAT4 observed in our study is consistent with their roles as downstream effectors of JAK activation, reflecting the enhanced inflammatory signaling characteristic of UC (32–34). STAT3, in particular, plays a key role in regulating immune responses and epithelial repair, but its persistent activation has also been associated with chronic inflammation and disease progression in UC (35).

The lack of a significant increase in JAK1 phosphorylation in biopsies from inflamed areas of the colon in our cohort is intriguing, particularly in light of the development of selective JAK1 inhibitors for UC treatment (36). This observation may reflect a high degree of variability in JAK1 activation among UC patients, underscoring the heterogeneous nature of the disease. Such variability highlights the potential importance of personalized approaches to UC management, where understanding individual signaling profiles could inform the selection of the most appropriate therapeutic strategies. These findings emphasize the need for further research to elucidate the factors influencing JAK1 activation and to assess their implications for tailored treatment in UC.

The observed phosphorylation levels within the JAK/STAT pathway provide a foundation for exploring the functional correlations among its components. These correlations offer further insights into the molecular dynamics underpinning UC-related inflammation.

The significant correlation between the phosphorylation of JAK2 and STAT3 (*r* = 0.534, *p <* 0.001) underscores the essential role of JAK2 in STAT3 activation (37). STAT3, a downstream effector of the JAK/STAT pathway, is a central mediator of IL-6 signaling and a key driver of tissue inflammation and repair dysregulation in UC (38). This correlation aligns with previous findings that STAT3 activation contributes to the severity of inflammatory processes underlying UC pathophysiology and is involved in the progression to colitis-associated cancer (35, 39). However, it is worth noting that STAT3 also exhibits a protective role by enhancing the production of mucus in the gut, adding a layer of complexity to its function in UC pathophysiology (40). Taken together, these results reinforce the notion that JAK2 serves as a central node in coordinating inflammatory pathways in UC.

Furthermore, there was a strong correlation between the phosphorylation of JAK2 and TYK2 (*r* = 0.545, *p <* 0.001) highlighting their synergistic roles in amplifying inflammatory signals, consistent with their established involvement in IL-12 and IL-23-mediated pathways (41, 42). TYK2, a kinase critical for the signaling of these cytokines, has been shown to work in concert with JAK2 to drive pro-inflammatory responses in various immune-mediated conditions (43). This synergy may reflect their roles in promoting the differentiation and activation of Th17 cells, which are pivotal in sustaining chronic inflammation in UC (44, 45).

Another layer of interconnection comes from the significant correlation between the phosphorylation of TYK2 and JAK3 (*r* = 0.539, *p <* 0.001), suggesting a functional interplay between these kinases in UC. As discussed before, JAK3 is known to participate in signaling pathways activated by cytokines such as IL-15, which are central to UC pathogenesis (16, 17). The correlation between the phosphorylation of TYK2 and JAK3 may indicate an interdependency in their activation, a relationship that, to our knowledge, has not been extensively explored. These findings suggest that further investigation into the interplay between these JAK kinases could provide novel insights into their roles in UC pathophysiology.

Another noteworthy observation is the significant correlation between the phosphorylation of effector proteins STAT1 and STAT3 (*r =* 0.429, *p =* 0.010), suggesting cooperative roles for these transcription factors in regulating inflammation. STAT1 and STAT3 are both downstream effectors of the JAK/STAT pathway and are activated by overlapping yet distinct cytokines. This interplay aligns with their shared involvement in mediating responses to pro-inflammatory cytokines such as IL-6, which is central to the pathogenesis of UC (12). IL-6 is known to lead to the activation of STAT3 (46), promoting inflammatory responses and impairing epithelial barrier function in UC (47). STAT1, typically activated by IFN-γ and other proinflammatory cytokines (33), may synergize with STAT3 under certain inflammatory conditions to amplify or modulate downstream transcriptional responses, contributing to the chronic inflammation characteristic of UC.

Taken together, all these correlations emphasize the complex and interdependent nature of JAK/STAT signaling in UC. The interactions among JAK2, JAK3, TYK2 and STAT3 likely reflect a coordinated effort to propagate and sustain inflammatory responses across multiple immune cell types.

Building on these findings, an analysis of phosphorylated JAK/STAT pathway components by sex revealed additional nuances in pathway activation. **Figure 4** highlights significant sex-dependent differences in JAK2 (*p =* 0.049) and STAT3 (*p =* 0.033) phosphorylation, while the remaining proteins showed no statistically significant variation. These results introduce a potential dependence on sex-specific factors in shaping the activation dynamics of the JAK/STAT pathway.

The higher median intensity of JAK2 phosphorylation in male patients compared to females suggests that JAK2 may play a more prominent role in propagating inflammatory responses in males. This finding aligns with evidence indicating that JAK2-mediated signaling is crucial for amplifying pro-inflammatory cytokines such as IL-6, which, as discussed before, plays a central role in UC pathogenesis (12). Similarly, the significant sex-dependent difference in STAT3 phosphorylation underscores its importance as a downstream effector, potentially reflecting sex-specific modulation of the IL-6/JAK2/STAT3 axis. This observation may correspond with prior reports of elevated IL-6 expression and activity in males across various pathological contexts, such as severe trauma and COVID-19, which are associated with exacerbated inflammatory responses (48, 49). Moreover, given the established association between STAT3 activation and an increased risk of developing colorectal cancer due to colitis, the observed increase in STAT3 phosphorylation in males may be linked to the higher incidence of colorectal cancer reported in male UC patients (50).

Although statistically significant sex-related differences were only observed for JAK2 and STAT3 phosphorylation, these findings are noteworthy given the moderate effect sizes and their achievement of statistical significance despite the relatively limited sample size. *Post hoc* power analyses indicated that the study may have been underpowered to detect small differences in the remaining pathway components (power range: 0.065–0.573; see **Table 4**). Consequently, the absence of statistical significance for other proteins should be interpreted with caution, and the observed trends in effect sizes may justify further investigation in larger, adequately powered cohorts. Overall, these results support the potential contribution of sex-related variability in JAK2 and STAT3 activation to differential immune responses in UC.

Taken together, these findings underscore the potential importance of considering sex as a biological variable in both research and treatment in inflammatory diseases, such as UC. Understanding these differences could pave the way for more personalized therapeutic strategies that account for sex-specific variations in pathway activation.

While the present findings provide valuable insights into JAK/STAT pathway activation in UC, certain aspects warrant cautious interpretation. Due to sample constraints, only the phosphorylated forms of each protein were analyzed, and total-protein levels were not assessed. Although phosphorylation serves as a widely accepted surrogate for activation, future studies incorporating both total and phospho-protein quantification could offer more comprehensive insights. Additionally, although we explored potential associations between phosphorylation levels and endoscopic disease activity, no significant correlations were identified in this cohort. This may be attributed to the limited sample size or variability in disease presentation, and future investigations in stratified or larger populations will be necessary to clarify these relationships.

In summary, this study offers a comprehensive analysis of JAK/STAT pathway activation in UC patients, emphasizing phosphorylation patterns and their functional correlations. Utilizing data from a real-world clinical cohort, we identified significant increases in the phosphorylation of six pathway components—JAK2, JAK3, TYK2, STAT1, STAT3, and STAT4—despite marked inter-patient variability in their activation. Notably, significant correlations between the activation of specific JAK kinases (JAK2, JAK3, TYK2) and STAT3 suggest a coordinated effort to amplify cytokine-driven inflammatory responses. Furthermore, the observed sex-specific differences, with enhanced activation of the JAK2/STAT3 axis in male UC patients, shed light on their elevated risk for colorectal cancer development. These findings reinforce the need to consider sex as a biological variable in therapeutic strategies to establish a foundation for developing personalized therapeutic approaches in inflammatory diseases.

## Data Availability

The original contributions presented in the study are included in the article/**Supplementary Material**, further inquiries can be directed to the corresponding authors.
