# Supplementary material for: Exploration of JAK/STAT pathway activation in ulcerative colitis reveals sex-dependent activation of JAK2/STAT3 in the inflammatory response
Source: Front Immunol. 2025 Jul 21;16:1609740. doi: 10.3389/fimmu.2025.1609740 (PMC12318950; doi:10.3389/fimmu.2025.1609740)
Supplement: Supplementary file 1 [file DataSheet1.docx]

Supplementary Material

# Supplementary Figures and Tables

## Supplementary Figures


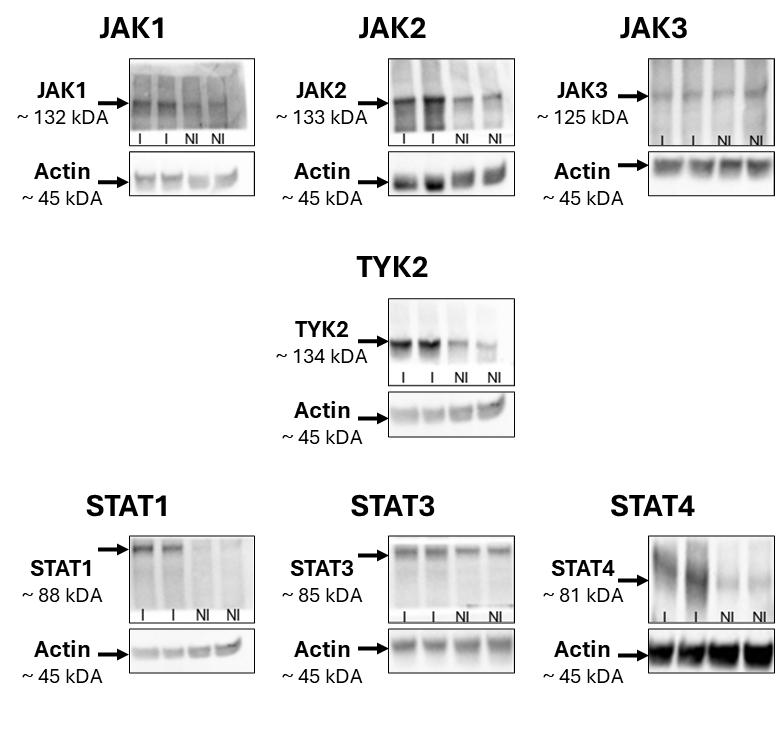


**Supplementary Figure 1.** **Representative Western blot images of phosphorylated JAK/STAT pathway components in colonic mucosa from UC patients.** Paired samples from inflamed (I) and non-inflamed (NI) regions of the colon were analyzed by Western blot using phospho-specific antibodies against JAK1, JAK2, JAK3, TYK2, STAT1, STAT3, and STAT4. β-actin was used as a loading control.
